# Stone’s Case of Uterine Mole

**Published:** 1848

**Authors:** R. R. Stone

**Affiliations:** Dorr, McHenry county, Illinois


### ARTICLE VI.

*Singular Case of Uterine Fleshy Mole.* By R. R. Stone, M.D., of Dorr, McHenry county, Illinois.

Mrs. H., Æt. 45, had been troubled with an enlargement of the uterus for some fifteen years. The distension commenced soon after a time when she considered herself pregnant, from the fact that there was a cessation of the menses at the time for some seven periods, but which again made their appearance, although quite irregular as to time and quantity. The uterus increased in size, rose in the pelvis, and seemed to go through the several stages of gestation with apparent motion of the child, until the seventh month when irregular pains commenced, and medical aid was called, but attempts to remove the then supposed dead fœtus did not succeed. She remained in that situation, partially recovering her health, but the distension remaining nearly the same, for six years, within which time she became pregnant and was delivered of a living child. During the period of gestation the enlarged uterus extended into the upper abdominal regions, and pressed hard upon the stomach and liver. After delivery the tumidity continued about as before pregnancy. Catamenia still irregular until last fall, when Dr. Coon, my partner, visited the patient. October tenth, by his request I saw her with him. She then presented the following symptoms. The uterus, which was enlarged to about the size of one in the seventh or eighth month of gestation, occupied principally the middle and right lower abdominal regions, the right side of the fundus being very hard and tender upon pressure. She suffered continually from pain which was mostly over the region of the tumor, but extended to the loins and down the thighs. But little could be ascertained concerning the nature of the contents of the uterus by examination per vaginum, as the os uteri was rigid, nearly closed, and above the superior strait. A small quantity of saneous and purulent fluid escaped during most of the time, together with small fragments of oscific matter resembling caries from the size of the finger nail to mere powder. Small fleshy masses were also frequently passed, some weighing over an ounce. As the patient was extremely weak, anodynes and tonics were administered.

But little relief was at first obtained. After a time, however, she began to convalesce, and was able to be about the house and ride out occasionally, and remained so until April 30th when, after riding six or eight miles in a lumber wagon, the former symptoms returned with increased severity. She was then seen by Dr. C. and myself. Had irregular uterine pains which distressed her much, but which were allayed by opiates.. The os uteri still remained rigid. An ointment of belladona was applied to the cervix which dilated the os tincæ in a measure. The pains, which were now permitted to go on, continued about 48 hours, when a soft, apparently fleshy mass, which presented at the superior strait, was finally, with manual assistance, expelled, when nearly a gallon of semi-purulent fluid escaped, together with so much fœtor that it was almost impossible to remain in the room. The substance expelled, upon examination, was found to consist of a homogenous conglobate mass, weighing about four and a half pounds, of a yellowish color, resembling healthy liver in consistence, but much more elastic and covered externally with scales of ossific matter. It was about ten inches in length, four in width, and from two to three in thickness, being somewhat wedge-shaped. Upon making an incision longitudinally a cartilaginous line was found to run transversely, and another to cross this at right angles, running the whole length.

The uterus contracted but partially at first, but it continued to contract and descend, so that at present it has assumed nearly its natural position, but is considerably enlarged, which condition I presume will remain. The ossific scales have continued to pass and are occasionally discharged at the present time. The fluid discharge continues, but in much less quantity. The patient is now able to walk a half mile, and is daily improving.

[The great length of time the tumor above described seems to have remained in the uterus, and the conception and delivery of a living child during the period, are very remarkable features in the case; the first of which renders it impossible to determine whether the mole was the result of conception or not, as changes might have taken place in the course of fifteen years, destroying all traces of the ovum had one existed at first. The attachment of the mass to one side of the uterus must have been quite firm at the time of the delivery of the living child, or the parturient efforts would have detached and expelled it.

The ossific scales that were formed on the surface of the mass, form another singular feature of the case, for which no satisfactory explanation can be given.

Altogether, it is one of the most singular cases on record.

